# Risk of hospital insolvency and its relationship with income and borrowings from banks: a case–control study with large-scale financial data in Japan

**DOI:** 10.1007/s43546-021-00153-7

**Published:** 2021-10-22

**Authors:** Satoshi Tsuboi, Tomosa Mine, Tetsuhito Fukushima

**Affiliations:** 1 Department of Hygiene and Preventive Medicine, Fukushima Medical University, 1 Hikarigaoka, Fukushima, 960-1295 Japan; 2 Faculty of Comprehensive Human Science, Shokei Gakuin University, Natori, Miyagi Japan

**Keywords:** Hospital, Financial distress, Hospital insolvency, Hospital management, Financial management, Finance, G33 bankruptcy, Liquidation

## Abstract

Considering the variety of stakeholders surrounding hospitals, hospital financial distress should be understood as a social issue, rather than just a matter involving the hospital owners. The present study aimed to assess Japanese hospital insolvency and related factors based on a nationwide financial dataset, and to identify indicators of the risk of insolvency. The legal financial reports used included a balance sheet and a profit-and-loss statement of hospitals owned by healthcare corporations, representing about 70% of all Japanese hospitals. This case–control study with descriptive analyses was conducted to clarify the financial status of healthcare corporations and to assess associations between specific factors and insolvency. Insolvency was found in 5.9% of healthcare corporations in 2016. Insolvency was significantly associated with operational income per sales (odds ratio, 0.16), and both short- and long-term borrowings per sales (odds ratios: 1.46 and 1.22 in this order). The present study found that 5.9% of Japanese healthcare corporations were insolvent, and hospital profitability and borrowing (both short- and long-term) could be key factors related to preventing hospital insolvency in Japan. To maintain sustainable healthcare services by hospitals, decision makers should consider the risk of insolvency, and balance the amount of borrowings against sales.

## Introduction

One of the major concerns in recent healthcare management is financial distress among hospitals and subsequent unwilling hospital closure, which affects a wide range of stakeholders, including patients. Hospital closures create potential barriers to accessing emergency services in a timely manner (Miller et al. 2020), may force patients in rural areas to drive longer to access services (McCarthy et al. 2021), and could disproportionately affect racial and ethnic minorities (Thomas et al. 2016). In a community, the volume of patients in hospitals neighboring closed hospitals typically increases (Lee et al. 2015; McKay and Dorner 1996), and the closure of a hospital may also reduce per-capita income and increase the unemployment rate (Holmes et al. 2006). Hospital financial distress should, thus, be understood as a social issue, rather than simply a matter involving hospital owners.

Although the number of studies is less than in other study areas, factors associated with hospital closures have been studied. The possibility of predicting hospital closures up to 2 years in advance of closure was reported (Lynn and Wertheim 1993). Financial data from hospitals, including profitability, liquidity, equity, and certain borrowings from banks are often used in studying and making such predictions (Lynn and Wertheim 1993; Kaufman et al. 2016). The index of financial distress for hospitals has recently been introduced as a good measure to understand the financial status of a hospital, comprising: (1) unprofitability; (2) declining equity; (3) insolvency; and (4) closure (Holmes et al. 2017). Along with this index, as the proximate financial distress to the hospital closure, insolvency is thought to be suitable to prevent hospital closures before they happen. (Although bankruptcy exists between hospital insolvency and closure, studies of bankruptcy, as well as hospital closure, tend to be difficult because this state cannot be defined from financial data (Holmes et al. 2017).) However, even though scientific research is expected to facilitate more effective financial management of hospitals, some Japanese hospitals have gone into financial distress with incredible levels of borrowing from banks (e.g., a Japanese hospital owned by a healthcare corporation went into bankruptcy with more than 40 million dollars in borrowings in 2019 (Teikoku Data Bank 2020)).

To the best of our knowledge, the present study is the first large-scale quantitative analysis to examine the financial status of Japanese hospitals. In Japan, research into hospital management, hospital financial management in particular, has been quite limited until now because the usage of hospital financial data is primitive. Legal reports on hospital financial status have been stored with local governments as papers, not in digital form. In addition, the cost of a commercial digital dataset of this financial data is so huge that large-scale quantitative research on this topic could not be conducted easily.

The present study aimed to assess Japanese hospital insolvencies and related factors. Considering previous studies, hospital profitability also seems likely to correlate negatively with hospital insolvency in Japan. In addition, borrowings from banks are hypothesized to be associated with hospital insolvencies. The present study offers a large-scale quantitative evaluation of the risk of hospital insolvency with financial data collected nationwide and indicators of the risk of insolvency. We hope that the present results will help decision makers (e.g., hospital managers, hospital owners, and other stakeholders) in scientifically assessing the risk of hospital insolvency and, thus, taking steps to prevent actual hospital closures.

## Materials and methods

With a grant from the Ministry of Education, Culture, Sports, Science and Technology (Japan), a dataset for all hospitals owned by healthcare corporations in 2016 was used in this study. According to the Survey of Medical Institutions by the Ministry of Health, Labour and Welfare (Japan), about 70% of all Japanese hospitals (5754 of 8442 hospitals in 2016) were owned by healthcare corporations that have an obligation to report their financial status, including a balance sheet and a profit-and-loss statement, to local governments every year (Ministry of Health, Labour and Welfare (Japan) 2017). In Japan, hospitals are defined as medical facilities containing over 20 beds for patients. Therefore, in this study, we collected the data for medical facilities with over 20 beds (i.e., hospitals) that have been owned by healthcare corporations.

Of the 5754 hospitals owned by healthcare corporations in 2016, we obtained characteristics that had been submitted to local governments by May 2019 (including information on the number of beds) for 5625 hospitals owned by 4723 healthcare corporations. After obtaining the data, we set the following inclusion criteria to check the integrity of the data: (1) total assets are equal to total liabilities and equity; (2) total current assets are not zero or missing; (3) total fixed assets are not zero or missing; (4) tangible fixed assets are not zero or missing; (5) total fixed assets are equal to the sum of tangible fixed assets, intangible fixed assets, and other assets; (6) total assets are equal to the sum of current assets and total fixed assets; (7) total liabilities are equal to the sum of current liabilities and fixed liabilities; (8) total liabilities and equity are equal to the sum of total liabilities and total equity; (9) sales are not zero or missing; (10) operational income is equal to sales minus costs and expenses; (11) income before taxes is equal to the sum of operational income and other income (expenses); (12) net income is equal to income before taxes minus provision for income taxes; (13) the number of beds is over 20; and (14) the types of beds are clearly mentioned. After this validation, 3707 healthcare corporations remained.

We conducted a descriptive analysis to determine quantitative aspects for owners of Japanese hospitals. A case–control study was also conducted to assess associations between hospital insolvency and other factors. Any healthcare corporations showing total equity less than zero was defined as insolvent. After quantitative description of the owners of Japanese hospitals with the whole dataset, cases (insolvent healthcare corporations) were matched with controls (other healthcare corporations) by the type and number of beds of hospitals. One case was matched with one control to adjust for confounding effects of the type and number of beds on hospital insolvency.

As a descriptive analysis, 25th percentiles, medians, and 75th percentiles of financial characteristics were stratified by three categories of the number of beds: 20–99, 100–199, and ≥ 200. Associations between categories of the number of beds and financial characteristics (sales, operational income, net income, total assets, current assets, fixed assets, total liabilities, short-term borrowings, long-term borrowings, equity) and additional indices (operational income per sales, net income per sales, total liabilities per sales, short-term borrowings per sales, and long-term borrowings per sales) to adjust for differences in economic scales of healthcare corporations were tested using the Kruskal–Wallis test. In addition, the chi-square test was used to assess relationships between hospital insolvency and liabilities (total liabilities per sales, short-term borrowings per sales, and long-term borrowings per sales). The Wilcoxon rank-sum test, Kruskal–Wallis test, and chi-square test are categorized as nonparametric alternatives. These statistical tests are useful, as they do not assume any particular distributions involving normal distribution. When two groups are used to compare continuous variables, the Wilcoxon rank-sum test should be chosen. Conversely, the Kruskal–Wallis test is chosen when three or more groups are used to compare continuous variables, and the chi-square test is chosen when three or more groups are used to compare binary variables (e.g., Insolvent vs. Non-insolvent). After making matched pairs of healthcare corporations as cases and controls one by one with the type and number of beds, associations between hospital insolvency and indices of profit (operational income per sales, and net income per sales) and liabilities (total liabilities per sales, short-term borrowings, and long-term borrowings) were tested using the Wilcoxon rank-sum test. As multivariate analysis in matched pairs, conditional logistic regression analysis was conducted to obtain odds ratios for hospital insolvency. Profit indices were included in Model 1. In Model 2, operational income per sales and net income per sales, as factors significantly associated with hospital insolvency in Model 1, were added with total liabilities per sales. In Model 3, both short-term borrowings and long-term borrowings per sales were entered into the model. Conditional logistic regression analysis is one of the multivariate analyses used to evaluate associations between an objective variable (binary) and explanatory variables. This analysis is useful to analyze the association between hospital insolvency and various financial indices in a model with matched pairs of samples. For outliers of total liabilities, short-term borrowings, long-term borrowings, and sales, the Smirnov–Grubbs test was applied to define outliers (Frank 1969). Since the Smirnov–Grubbs test assumes a normal distribution, we used the results from this test as reference. We have, therefore, shown the results of Models 1, 2, and 3 of all matched pairs of healthcare corporations and the results of these same models without outliers.

In this study, a value of P < 0.05 was considered statistically significant. All statistical analyses were conducted using STATA for Mac version 15.1 (Stata Corp, College Station, TX, USA).

## Results

Table 1 shows the characteristics of Japanese healthcare corporations according to the number of beds. Beds for psychiatric treatment tended to be more frequent in healthcare corporations with over 200 beds. In all three bed-number categories, at least 25% of healthcare corporations had a negative operational income, while at least 25% of healthcare corporations had no short-term borrowings. On the other hand, compared to the other characteristics, missing data were more frequent in both types of borrowings: 889 for short-term borrowings, and 363 for long-term borrowings. This might be explained as data being handled as missing in the dataset if borrowings were not mentioned in the balance sheet, instead of being written as zero. Median sales were 7939 thousand dollars for healthcare corporations with under 99 beds, 14,650 (1.8 × the 7939) in healthcare corporations with 100–199 beds, and 26,455 (3.3 × the 7939) in healthcare corporations with over 200 beds. Median operational income per sales was 1% in healthcare corporations with under 99 beds, followed by 2% in healthcare corporations with 100–199 beds, and also 2% in healthcare corporations with > 200 beds, and the same results were seen for net income per sales.

**Table 1:** Characteristics of Japanese healthcare corporations, stratified by number of beds

Percentiles	≤ 99 (*n* = 1611)			100–199 (*n* = 1160)			≥ 200 (*n* = 936)			*p* value
	25	50	75	25	50	75	25	50	75	
Number										
(Bed type)										
General	0	38	52	0	48.5	100	0	0	194.5	–
Recuperation	0	0	46	0	48	98	0	0	144.5	
Psychiatric	0	0	0	0	0	0	0	168.5	274	
Thousand dollars										
Sales	5106	7939	12,618	9568	14,650	22,902	17,155	26,455	43,954	< 0.01
Operational income	−133	101	450	−162	230	780	−85	604	1574	< 0.01
Net income	−89	95	372	−92	224	648	4	460	1292	< 0.01
Current assets	1659	2972	5430	3597	5989	9511	7131	11,780	20,445	< 0.01
Fixed assets	2401	4879	9075	5860	10,366	17,516	12,377	20,537	38,005	< 0.01
Total assets	4611	8452	14,651	10,449	16,727	27,068	21,181	33,998	57,206	< 0.01
Total liabilities	1379	3488	7232	3190	7580	15,734	6204	14,677	30,773	< 0.01
Short-term borrowings	0	282	903	0	571	1905	0	1143	4030	< 0.01
Long-term borrowings	633	2278	5404	1731	5039	11,413	3582	9933	21,377	< 0.01
Equity	1284	3602	7531	3200	6900	13,494	7651	15,449	29,269	< 0.01
Percent (per sales)										
(Per sales)										
Operational income	−2	1	5	−1	2	5	0	2	5	< 0.01
Net income	−1	1	4	−1	2	4	0	2	5	< 0.01
Total liabilities	22	44	77	25	51	82	29	53	84	0.01
Short-term borrowings	0	4	11	0	4	11	0	4	11	0.75
Long-term borrowings	10	29	58	13	35	62	15	36	61	0.01

Exchange rate: $1 = 105 yen

Missing: 889 short-term borrowings, and 363 long-term borrowings

In this table, there are 3 major columns (less than 99 beds group, 100–199 beds group, and more than 200 beds group), each of which is divided into 3 categories (25th percentile, 50th percentile, and 75th percentile). Because various patterns of bed type in a hospital are seen, the number of beds shown in this table might be less than the number in its category. For example, the number of general-type beds in the 50th percentiles of category 100–199 is 48.5, which is less than 100

Table 2 shows the number and percent of insolvencies by total liabilities per sales, short-term borrowings per sales, and long-term borrowings per sales. Insolvency was found in 5.9% of all healthcare corporations. In addition, insolvency was significantly associated with each of total liabilities per sales, short-term borrowings per sales, and long-term borrowings per sales. The frequency of insolvencies increased as each of these three indices increased. The frequency of insolvency was highest (21%) in healthcare corporations with long-term borrowings per sales over 0.9.

**Table 2:** Number and percent of insolvencies by total liabilities per sales, short-term borrowings per sales, and long-term borrowings per sales

	Number	Insolvent	Percent	*p* value
Total liabilities per sales				
< 0.3	1161	3	0.3	< 0.01
≥ 0.3, < 0.6	1067	23	2	
≥ 0.6, < 0.9	758	51	7	
≥ 0.9	721	140	19	
Short-term borrowings per sales				
< 0.03	1289	44	3	< 0.01
≥ 0.03, < 0.06	401	33	8	
≥ 0.06, < 0.09	274	24	9	
≥ 0.09	854	96	11	
Long-term borrowings per sales				
< 0.3	1588	33	2	< 0.01
≥ 0.3, < 0.6	921	44	5	
≥ 0.6, < 0.9	500	54	11	
≥ 0.9	335	72	21	

The “number” column means the number of healthcare corporations that fit into a feature shown in the left column. For example, this table shows that 1588 healthcare corporations had less than 0.3 long-term borrowings per sales: of these, 33 (2%) were insolvent

Table 3 shows the difference between insolvent healthcare corporations and others, after matching for the type and number of beds. Median operational income per sales and median net income per sales of insolvent healthcare corporations were negative and significantly lower than those of other healthcare corporations. Conversely, total liabilities per sales and long-term borrowings per sales were significantly higher for insolvent healthcare corporations than for other healthcare corporations.

**Table 3:** Difference between insolvent healthcare corporations and others after matching for type and number of beds

	Insolvent (*n* = 204)	Non-insolvent (*n* = 204)	*p* value
Median, thousand dollars			
Sales	8917	11,221	0.06
Median, percent			
Operational income per sales	−2	2	< 0.01
Net income per sales	−3	2	< 0.01
Total liabilities per sales	102	42	< 0.01
Short-term borrowings per sales	3	8	< 0.01
Long-term borrowings per sales	73	30	< 0.01

This table shows two major columns (insolvent and non-insolvent), and medians of financial factors listed in the left side are shown for each column. For example, median sales for insolvent healthcare corporations were 8917 (thousand dollars), and median operational income per sales in non-insolvent hospitals was 2%

Table 4 shows results from conditional logistic regression analysis with healthcare corporations matched for the type and number of beds. Within sales itself, operational income per sales, and net income per sales, operational income per sales and net income per sales showed significant negative associations with hospital insolvency in Model 1 (odds ratio, 0.42 and 0.55 in this order). Total liabilities per sales were significantly and positively correlated with hospital insolvency after adjusting for the effect of operational income per sales and net income per sales in Model 2 (odds ratio, 1.32). Moreover, both short- and long-term borrowings per sales were positively associated with hospital insolvency after adjusting for the effect of operational income per sales and net income per sales in Model 3 (odds ratios: 1.46 and 1.22, respectively). Associations between insolvency and both of these borrowings were significant and independent. As outliers, 27 samples were eliminated from the original matched pairs of samples. The results after eliminating data outliers were not essentially different from the original results.

**Table 4:** Odds ratios and 95% confidence intervals for insolvency

	Model 1	Model 2	Model 3
All samples			
Sales (units: 10,000,000 dollars)	1.00 (0.97–1.02)	–	–
Operational income per sales (units: 10 percent)	0.42 (0.25–0.69)*	0.32 (0.16–0.62)*	0.16 (0.07–0.37)*
Net income per sales (units: 10 percent)	0.55 (0.34–0.88) *	0.75 (0.43–1.31)	1.18 (0.77–1.81)
Total liabilities per sales (units: 10 percent)	–	1.32 (1.21 – 1.44)*	–
Short-term borrowings per sales (units: 10 percent)	–	–	1.46 (1.05–2.03)*
Long-term borrowings per sales (units: 10 percent)	–	–	1.22 (1.11–1.34)*
Sample outliers eliminated			
Sales (units: 10,000,000 dollars)	0.99 (0.82–1.20)	–	–
Operational income per sales (units: 10 percent)	0.41 (0.24–0.68)*	0.31 (0.16–0.61)*	0.14 (0.06–0.35)*
Net income per sales (units: 10 percent)	0.59 (0.36–0.94) *	0.78 (0.45–1.35)	1.26 (0.81–1.94)
Total liabilities per sales (units: 10 percent)	–	1.29 (1.19–1.41)*	–
Short-term borrowings per sales (units: 10 percent)	–	–	1.63 (1.09–2.43)*
Long-term borrowings per sales (units: 10 percent)	–	–	1.18 (1.08–1.30)*

Numbers in parentheses represent 95% confidence intervals

The common objective variable for these three models (Model 1, Model 2, and Model 3) is insolvency, and the explanatory variables are varied depending on the models. Explanatory variables in Model 1 are sales, operational income per sales, and net income per sales. Explanatory variables in Model 2 are operational income per sales, net income per sales, and total liabilities per sales. Explanatory variables in Model 3 are operational income per sales, net income per sales, short-term borrowings per sales, and long-term borrowings per sales. Results of Model 1, Model 2, and Model 3 for all matched pairs of healthcare corporations and the results of these same models without outliers are shown

*Significant results

## Discussion

In the present study, financial status in Japanese hospitals owned by healthcare corporations was examined with a large-scale quantitative analysis. In Japan, 5.9% of healthcare corporations were insolvent in 2016. Borrowings from banks, both short- and long-term, seem to play an important role in preventing insolvency. Of note, operational income per sales rather than sales itself was associated with insolvency. This finding of the importance of profitability in Japanese hospital management is in line with findings from other countries (Kaufman et al. 2016; Bai et al. 2020; Holmes et al. 2013; Ly and Cutler 2018; Pai et al. 2017). Likewise in Japan, stakeholders such as hospital managers or owners, bankers, and policy-makers should focus on the financial status of hospitals for providing sustainable healthcare services and securing the local economy (Miller et al. 2020; McCarthy et al. 2021; Thomas et al. 2016; Holmes et al. 2006).

In general, two completely different methods are available for improving profitability: increasing sales or decreasing cost. According to previous studies (Bai et al. 2020; Ly and Cutler 2018; Pai et al. 2017), increasing sales by improving quality of care is considered better than cost reduction. Dan et al. showed that hospitals that became more profitable were more likely to increase admissions per bed per year, and showed a larger magnitude of increases in revenue per bed than decreases in costs per bed (Ly and Cutler 2018). In addition, critical-access hospitals (Holmes et al. 2013), not-for-profit status (Ly and Cutler 2018), and joining a hospital system are known to be positively associated with hospital profitability (Ly and Cutler 2018; Büchner et al. 2016; O'Hanlon et al. 2019), although affiliation may reduce access to services for patients in rural areas (O'Hanlon et al. 2019). Furthermore, nurses and doctors are crucial to maintain the quality of healthcare services provided at hospitals (Pai et al. 2017; Boakye et al. 2019; Tasi et al. 2019). Interpersonal support and supervisory support are positively associated with nurse/employee engagement and their retention (Boakye et al. 2019). Physician-led hospital systems showed higher quality ratings than non-physician-led hospital systems (Tasi et al. 2019). Although these are supportive findings for improving quality of care in hospitals, managerial decision-making for increasing profitability is still difficult due to insufficient quantitative evaluation of the relationship between quality of care and hospital profitability. For example, effective methods to accomplish a 10% increase in profitability (shown in the present results) remain uncertain. Methods of improving quality of care in hospitals, following hospital profitability, need to be studied in greater detail to improve hospital sustainability.

Borrowings (especially long-term borrowings) per sales seem to offer good indicators of the risk of insolvency; a 10% increase in long-term borrowings per sales represented a 22% increase in the risk of insolvency. It is an appropriate financial status that a repayment is covered by sales within the same period, or new borrowings are planned according to expected sales. Calculating future sales is thought to be easier than other industries, at least in Japan, because the number of beds is totally under the control of the local government (Zhang and Oyama 2016; Hosokawa et al. 2020). Opening a new hospital is not permitted by local governments unless the actual number of beds is less than the governmental expected number calculated to meet medical demand in that area. That is, competition between hospitals is strictly limited to stabilize healthcare service supply. In addition, considering that the amount of borrowings is directly controlled by managerial decision-making, Japanese hospitals can thus make reasonable plans for borrowings for existing business with reference to the relatively stable expected sales. On the other hand, uncertainty increases when planning a new business, such as constructing a huge dialysis center in a local setting, as well as under global pandemics of infectious diseases, including coronavirus disease 2019. Previous financial management is thought to play a major role in coping with difficulties arising from such uncertain situations. In hospital financial management, maintaining a balance between sales and borrowings is essential to prevent hospital insolvency. At this point, the results of the present study about borrowings are useful as indicators to control the amount of borrowings in hospitals.

A few limitations to this investigation should be noted. First, the hospitals analyzed in this study were limited to those owned by healthcare corporations in Japan. Although the proportion of healthcare corporation-owned hospitals among all Japanese hospitals was reported as about 70% (Ministry of Health, Labour and Welfare (Japan), 2017), application of the results of this study to public hospitals or hospitals owned by individuals needs careful consideration. Second, a case–control study conducted with data from 2016 limits the insights into causal relationships between hospital insolvency and other factors. The last limitation is the validity of data reported by hospitals to local governments without necessarily requiring objective checks by authorized individuals. The criteria to select samples in this study resulted in a loss of 22% of total data. The results of this study should, thus, be confirmed in future studies, including longitudinal studies with data from other years.

## Conclusions

The present study found that 5.9% of Japanese healthcare corporations were insolvent, and hospital profitability and borrowing (both short- and long term) could be key factors related to preventing hospital insolvency in Japan. Although effective manners to increase hospital profitability are still unclear, the amount of borrowings is directly controlled by managerial decision-making. To maintain sustainable healthcare services by hospitals, decision makers should consider the risk of insolvency, and balance the amount of borrowings against sales.

## Data Availability

Although the data used in this study are obtainable from Japanese local governments, the dataset used will not be available, since data-sharing is prohibited by the contract with the data vendor.

## References

[CR1] Bai G, Yehia F, Chen W, Anderson FG (2020). Varying trends in the financial viability of US rural hospitals, 2011–17. Health Affairs (project Hope).

[CR2] Boakye GK, Apenteng AB, Hanna DM, Kimsey L, Mase AW, Opoku TS, Owens C, Peden A (2019). The impact of interpersonal support, supervisory support, and employee engagement on employee turnover intentions: differences between financially distressed and highly financially distressed hospitals. Health Care Manag Rev.

[CR3] Büchner AV, Hinz V, Schreyögg J (2016). Health systems: changes in hospital efficiency and profitability. Health Care Manag Sci.

[CR4] Frank EG (1969). Procedures for bes. Technometrics.

[CR5] Holmes MG, Slifkin TR, Randolph KR, Poley S (2006). The effect of rural hospital closures on community economic health. Health Serv Res.

[CR6] Holmes MG, Pink HG, Friedman AS (2013). The financial performance of rural hospitals and implications for elimination of the Critical Access Hospital program. J Rural Health.

[CR7] Holmes MG, Kaufman GB, Pink HG (2017). Predicting financial distress and closure in rural hospitals. J Rural Health.

[CR8] Hosokawa R, Ojima T, Myojin T, Aida J, Kondo K, Kondo N (2020). Associations between healthcare resources and healthy life expectancy: a descriptive study across secondary medical areas in Japan. Int J Environ Res Public Health.

[CR9] Jansen OJ (2021). Impact of rural hospital closures on health-care access. J Surg Res.

[CR10] Kaufman GB, Thomas RS, Randolph KR, Perry RJ, Thompson WK, Holmes MG, Pink HG (2016). The rising rate of rural hospital closures. J Rural Health.

[CR11] Ministry of Health, Labour and Welfare (Japan) (2017) The survey of medical institutions. https://www.mhlw.go.jp/toukei/saikin/hw/iryosd/16/. Accessed 27 May 2021 **(in Japanese)**

[CR12] Lee CD, Carr GB, Smith ET, Tran CV, Polsky D, Branas CC (2015). The impact of hospital closures and hospital and population characteristics on increasing emergency department volume: a geographic analysis. Popul Health Manag.

[CR13] Ly PD, Cutler MD (2018). Factors of US hospitals associated with improved profit margins: an observational study. J Gen Intern Med.

[CR14] Lynn LM, Wertheim P (1993). Key financial ratios can foretell hospital closures. Healthc Financ Manag.

[CR15] McCarthy S, Moore D, Smedley AW, Crowley MB, Stephens WS, Griffin LR, Tanner CL, Jansen OJ (2021). Impact of rural hospital closures on health-care access. J Surg Res.

[CR16] McKay LN, Dorner HF (1996). The effect of rural hospital closures on the financial performance of neighboring rural hospitals. Inquiry.

[CR17] Miller EMK, James JH, Holmes MG, Houtven VHC (2020). The effect of rural hospital closures on emergency medical service response and transport times. Health Serv Res.

[CR18] O'Hanlon EC, Kranz MA, DeYoreo M, Mahmud A, Damberg LC, Timbie J (2019). Access, quality, and financial performance of rural hospitals following health system affiliation. Health Affairs (project Hope).

[CR19] Pai RD, Hosseini H, Brown SR (2017). Does efficiency and quality of care affect hospital closures?. Health Syst (basingstoke, England).

[CR20] Tasi CM, Keswani A, Bozic JK (2019). Does physician leadership affect hospital quality, operational efficiency, and financial performance?. Health Care Manag Rev.

[CR21] Teikoku Data Bank (2020) The survey of bankruptcy in the Japanese clinics and hospitals. https://www.tdb.co.jp/report/watching/press/p200101.html. Accessed 27 May 2021 **(in Japanese)**

[CR22] Thomas RS, Holmes MG, Pink HG (2016). To what extent do community characteristics explain differences in closure among financially distressed rural hospitals?. J Health Care Poor Underserved.

[CR23] Zhang X, Oyama T (2016). Investigating the health care delivery system in Japan and reviewing the local public hospital reform. Risk Manag Healthc Policy.

